# Creating unity: linking 16S rRNA gene sequence information to the core taxonomy of genomes

**DOI:** 10.1186/s40793-025-00789-0

**Published:** 2025-10-28

**Authors:** Hilde Vinje, Knut Rudi, Lars Snipen

**Affiliations:** Faculty of Chemistry, Biotechnology and Food Science, Norwegian University of Life Sciences (NMBU), P.O. Box 5003, Ås, NO-1432 Norway

## Abstract

**Background:**

The Genome Taxonomy Database (GTDB) initiative aims to modernize prokaryotic taxonomy by aligning it with the great amounts of full-length genomes available today. Unfortunately, there is a poor link between the GTDB and the historically widely used 16S rRNA gene-based taxonomy. The current study explores the within and between divergence of the 16S rRNA gene sequences under GTDB taxonomy, refining our understanding of the 16S gene’s resolution under this new taxonomic system. The analysis focuses on the divergence of 16S sequences collected from the GTDB genomes to identify optimal clustering thresholds for taxonomic resolution. Generalized linear mixed models (GLMMs) were fitted to estimate divergences within taxonomic ranks, correcting for the variable quality of the GTDB genomes.

**Results:**

To achieve GTDB species-level resolution, 16S sequences need clustering at a divergence threshold of around 0.01 (99% identity), while genus-level resolution requires thresholds of 0.04–0.08 (92–96% identity), optimal thresholds vary significantly across branches, highlighting the limitations of using a fixed divergence threshold.

**Conclusions:**

The results suggest a more adaptive approach to taxonomic assignment from 16S data is needed, tailored to the diversity and complexity of the samples. These findings are fundamental for an improved taxonomic classification of environmental 16S data.

**Supplementary Information:**

The online version contains supplementary material available at 10.1186/s40793-025-00789-0.

## Introduction

There is currently a conceptual shift in prokaryote taxonomy, from 16S rRNA gene [1] to whole genome-based taxonomies [2]. With the widespread use of 16S sequencing and classification today, a key feature will be to strengthen the connection between the two approaches: whole-genome and 16S rRNA gene-based taxonomies. There are clear challenges with the latter, as noted by Hassler et al. [3], but we do not believe that abandoning 16S rRNA gene sequencing is the solution. In environments outside the extremely well-defined human microbiome, traditional metagenome-based approaches can only pick up the tip of the iceberg, being severely biased towards knowledge from the human microbiome [3, 4]. We believe a more effective and rational approach would be to integrate 16S rRNA gene information with the latest whole-genome taxonomy, creating a unified platform where data from all technologies are interpreted within a shared taxonomic framework.

Countless studies are, partly or entirely, based on amplicon sequencing of the 16S gene (e.g [4–9]). Grouping these sequences by their similarity allows us to say something about the diversity, abundance and prevalence of various taxonomic units in the samples [10–13]. This is especially applicable when sampling new environments, where we cannot simply map reads to already known reference sequence variants. A fundamental piece of information required to obtain biologically meaningful groups is how much variation does this gene display within and between taxa at various ranks. We may cluster or denoise reads to obtain sequence variants from our data, but how many taxa will they represent? This information is crucial if we are to ensure the differentiation at various ranks, and it is a recurring theme when using 16S for taxonomic classification [14].

There is no official prokaryotic taxonomy [15]. Consequentially, the taxonomic assignments we encounter in the various 16S data repositories, e.g. NCBI [16], SILVA [17], RDP [18] and Greengenes [19], differ. Historically, a phenotype-based classification was used, gradually shifting to be more governed by 16S rRNA divergence in recent times [20].

With the growing amounts of whole genome sequence data, it seems natural to base the taxonomy on more than a single gene. This was the motivation behind the Genome Taxonomy Data Base project (GTDB [2]). By extracting suitable marker genes from hundreds of thousands of genomes, they have made an alternative taxonomy based on monophyletic groups obtained from such data. The GTDB taxonomy is now widely used for assigning taxonomy to genomes obtained from (meta)genome sequencing [2]. For the sake of consistency, it is also natural to then use the same taxonomy for 16S rRNA data. The ability to continue using 16S classification methodology, even with data derived from whole genomes, is a critical issue and must be thoroughly investigated and definitively established. Hence, being able to say something about the divergence of the 16S gene under the GTDB taxonomy, is of great interest.

In the current study we use the GTDB database to investigate how the genome-based taxonomy shed light on the 16S divergence within and between taxa at all ranks. We quantify 16S divergence by the use of a generalized linear mixed models to estimate and compensate for the various quality levels of the genomes as well as smoothing estimates across the taxonomy. We also provide a collection of the distinct GTDB 16S sequences for the use with tools where taxonomic classification or closed reference assignments require a comprehensive database. Due to the direct link between these 16S variants and their genomes it brings 16S data closer to the functional repertoire of the taxa. Our results have direct implications for ecological studies on taxonomic richness and diversity and reveal the expected resolution when working with 16S data within the GTDB taxonomy.

## Methods

### Data

From the GTDB website [2], release 2.26 (April 16, 2025), we downloaded all extracted 16S sequences (ssu_all_r226.fna). According to the GTDB documentation, these 16S sequences were extracted from the genomes using the HMMER3 software [21] (nhmmer tool) and the secondary structure correlation models for the bacteria (RF1700) and archaea (RF1959) from the Rfam database [22]. We discarded all 16S sequence shorter than 800 bases, i.e. keeping only sequences covering at least half the 16S gene. Then, each of the sequences were scanned by the infernal software [23] (cmsearch tool) using the correlation models mentioned above. Scanning a proper 16S sequence this way typically produces a bit-score of around the same size as its length. The resulting bit-score was divided by the sequence length, and sequences with a value below 0.9 were discarded as not reliable 16S sequences.

Each 16S sequence has an accession indicating the corresponding GTDB genome it has been extracted from. Hence, all meta data about the genomes were joined with the 16S sequences. This means they all have a full GTDB taxonomy. Since the GTDB genomes are all downloaded from NCBI in the first place, they also have a lot of other meta data, including the NCBI assembly level as a quality measure for each genome.

### Divergence

To compute the similarity between all pairs in a set of sequences we used the software vsearch [13] with the usearch global tool and using the same sequences as both query and database. This software is built for parallel processing 16S data and may align large numbers of sequence pairs fast. The algorithm scores mismatches and gaps at the ends close to neutral, producing essentially semi-local alignments. This adds robustness when comparing 16S sequences of unequal lengths. The sequence identity was computed as the fraction of identical bases in the alignment but excluding terminal gaps.

A measure of divergence between two 16S sequences is then simply 1 minus this identity. However, for statistical analysis we acknowledge that this divergence is better described as counts (integers) of differences between the sequences since such data have distributions typical of count data. Thus, for our analysis we used the divergence defined as

$$\:d=\left[1000\left(1-identity\right)\right]$$

where [.] means rounding to the nearest integer. This means *d* is the number of differences per 1000 bases of 16S sequence. We found that the factor 1000 produced values very close to integers, and the rounding is only needed to get actual integer count data as input to the functions used in the downstream statistical analysis. Even if we used these integers as divergence data, we still display divergence as a decimal value when reporting such results, e.g. instead of *d = 10* we show it as *10/1000 = 0.01*.

### Generalized linear mixed model

The genomes used by the GTDB all belong to one out of four *assembly levels* reflecting the quality of the genome sequence. The best quality level is Complete Genome. Ideally, all results we derive from genomes should be based on data from such genomes, but the majority of the genomes are from poorer quality levels (Chromosome, Scaffold or Contig). Many taxa have no genomes of type Complete Genome. In the text we sometimes refer to high-quality and low-quality genomes, the former being Complete Genome or Chomosome and the latter Scaffold or Contig genomes.

To estimate a count statistic like divergence we employ a Generalized Linear Mixed Model where the assembly levels are the fixed effects and the taxonomy is used as nested random effects. This allows us to estimate, and compensate for, any effect of the lower assembly levels and it also produces a smoothing across the taxonomy where information is “borrowed” from related taxa in the taxonomy tree. The idea is that effects tend to be more similar for taxa nested under the same taxon in the rank above (e.g. for all species under the same genus) and borrowing information from such neighbors will stabilize all estimates, especially for those taxa with few data. We may express the model as

$$\begin{aligned} g(y_{{idpcofgs}} ) = & \alpha _{i} + D_{d} + P_{{p|d}} + C_{{c|p}} + O_{{o|c}} \\ & + F_{{f|o}} + G_{{g|f}} + S_{{s|g}} + e_{{idpcofgs}} \\ \end{aligned} $$

where the function $g(.)$ is some link function in the generalized linear model, the *y* is the count (divergence), the *a*_*i*_ are the fixed effects of assembly level and the capital letters indicate the random effects of domain (*D*), phylum (*P*), class (*C*), order (*O*), family (*F*), genus (*G*) and species (*S*). The last term *e* is some random error term. Note that the random effects are nested, and to be correct we should write e.g. *S*_*s|g|f|o|c|p|d*_ but to simplify the notation we only condition on the rank above. The indices indicate each taxon under the parent taxon. All random effects are assumed normal distributed with expected value *0* and some variance, e.g. *σ*_*S*_^*2*^ is the variance for species, *σ*_*G*_^*2*^ for genus etc.

Since our data are counts, the error distribution may be Poisson, quasi-Poisson or negative binomial depending on the over-dispersion of the data. We tested all three, but used the simplest Poisson model unless there were clear evidence of over-dispersion based on the fitted models. All variants of this model was fitted to data using the glmmTMB R package [24].

The main reason for fitting the GLMM to the data was to predict (Best Linear Unbiased Predictor) the divergences for the Complete Genome (high-quality) assembly level only. In this way we get smoothed results for all taxa based on all data, but transformed into values reflecting what we expect to observe had all genomes been of the highest quality level. In addition, we also estimate the variances of the random effects. This indicates how similar/different related taxa at the various ranks in the taxonomy are to each other with respect to divergence.

### Clustering

Sequences were also clustered, for two reasons. First, we wanted to obtain the set of distinct sequences within species. This was achieved by computing all pairwise divergences between sequences within each species followed by a hierarchical clustering with complete linkage and then split into clusters at 0 divergence. We again used vsearch for the pairwise divergence computations as described above, and the rest was done with custom R code.

Second, we wanted to cluster these distinct sequences at various divergence thresholds in order to see how the clusters compare to the actual species and genera. For this we used only the distinct sequences from high quality genomes. First, the sequences were arranged by species and then split into 10 equal-sized non-overlapping subsets. This means the taxonomic composition of each subset is unique. Each subset was then clustered at various divergence thresholds. For this we used vsearch with the–cluster_size option to simply group sequences by a given identity/divergence threshold. The clusters were then compared to the ‘true’ grouping of the sequences by either species or genus.

Second, we organized the same sequences by phylum, resulting in 65 non-overlapping subsets. This means the number of taxa and sequences varies between the subsets. The same clustering and comparison to actual grouping by species and genus was employed.

## Results

From the GTDB website we downloaded the file ssu_all_r226.fna containing all 16S sequences provided by the GTDB release 2.26. Sequences shorter than 800 bases were discarded. We also discarded sequences with a too low bitscore after comparing them to the secondary structure models for 16S rRNA. This resulted in a total of 803 505 near full-length 16S sequences in the data set.

The current version of the GTDB taxonomy is based on 732 475 genomes. The 16S data come from 408 679 of these, i.e. there are more than 320 000 genomes (44%) without any 16S of at least 800 bases with an Infernal score below 0.9. The 16S data cover in total 61 313 species in 15 998 genera. This includes 3 002 archaeal and 58 311 bacterial species. All the GTDB-genomes are categorized into four quality levels (the NCBI assembly-levels Complete Genome, Chromosome, Scaffold and Contig) and as shown in Table 1, most genomes are of the two poorest quality categories (Scaffold and Contig). From this table we also notice that in these two categories a lot of genomes lack 16S since the numbers in the second column in Table 1 are much smaller than in the first column. When we count how many 16S sequences we have from genomes of the various qualities, we see that Complete Genomes contribute with a lot. This is because such genomes often have multiple copies of 16S while Scaffold and Contig genomes most typically has only one.

**Table 1:** GTDB genome quality distribution and 16S sequences

Genome quality levels	Genomes	Genomes with 16S	16S sequences
Complete Genome	49 324	49 223	262 225
Chromosome	6 750	6 038	24 158
Scaffold	247 548	125 687	164 557
Contig	428 853	227 731	340 235

Under Genome is listed the total number of genomes of the four quality levels. The column Genomes with 16S is the similar count for those genomes with a 16S of at least 800 bases. Finally, the 16S sequences column lists the total number of 16S sequences from genomes of the four quality levels

### Within genome divergence

**Fig. 1:**
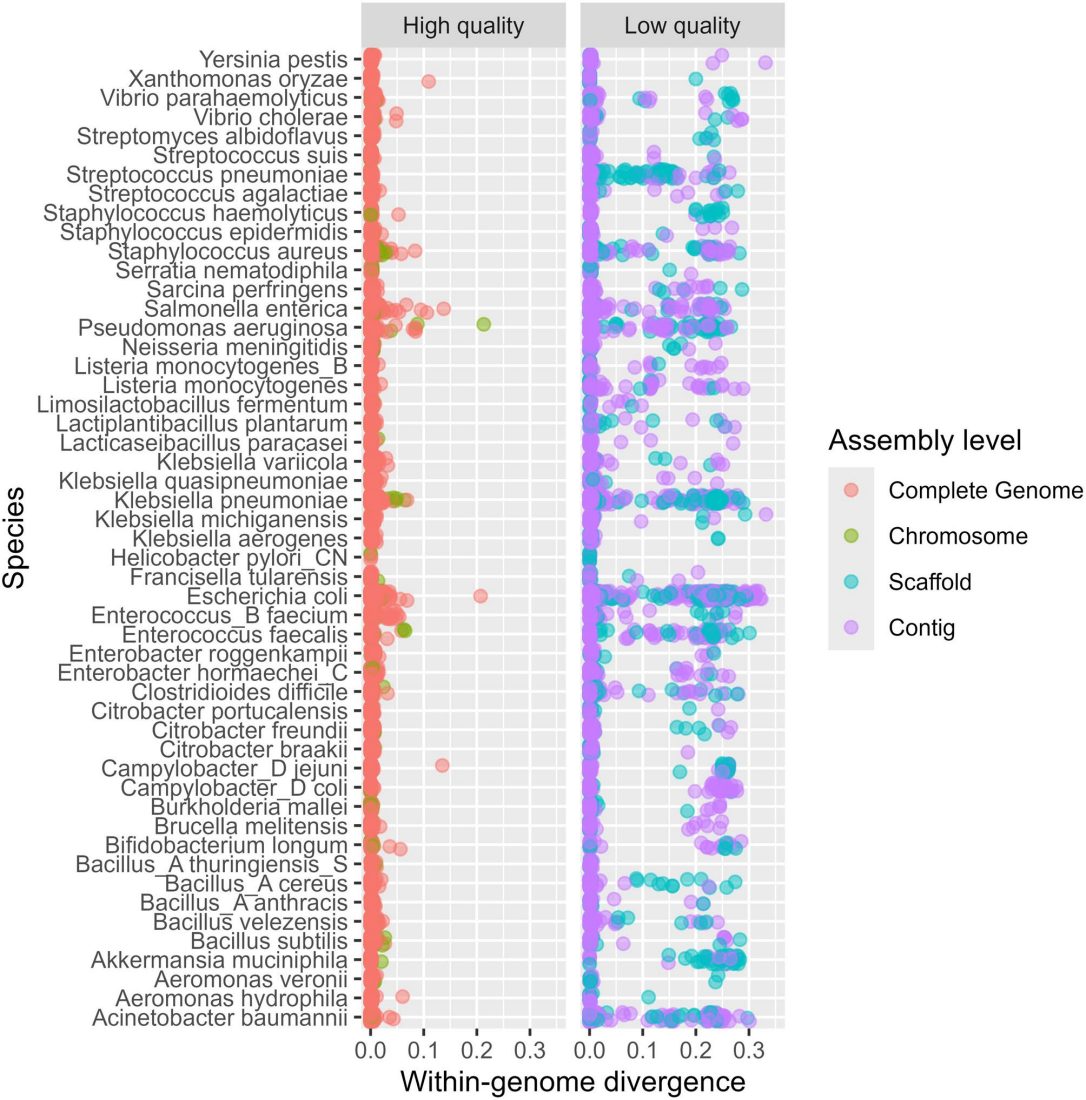
Each dot is the mean within-genome divergence for a genome of some quality level and for a selection of species. These species were selected because they have at least 5 genomes at all four quality levels. The left panel show divergences (x-axis) for high-quality genomes and the right panel similar for low-quality genomes

The ‘multiple copies’ of 16S within a genome is a misnomer since even within a single genome we frequently find two or more variants of 16S. In Fig. 1 we show the mean within-genome divergence between 16S variants for some selected species. This also demonstrates why it is important to account for the genome quality in such studies. For the high-quality genomes (left panel) these within-genome divergences are usually small, while for the low-quality genomes (right panel) we sometimes find huge differences between the 16S variants inside the same genome.

Based on this observation, we estimated the within-genome divergences based only on high quality genomes (assembly levels Complete Genome or Chromosome). For all such genomes with two or more 16S copies, we fitted a GLMM using mean within-genome divergence per genome as the response variable, assembly level as a fixed effect, and taxonomic ranks as nested random effects. This data set contains more than 830 000 divergences covering 10 169 species. A negative binomial family (nbinom2) was used due to over-dispersion in the data. From this we predicted within-genome divergences for a Complete Genome across all species. The first column in Table 2 lists some quantiles of the distribution of these predictions. These values serve as a guide to determining the level of stringency required when clustering 16S sequences to be confident that variants from the same genome fall into the same cluster. The median (50% quantile) is an estimate of the expected within-genome divergence, and this is very close to zero for Complete genomes. However, to have a 99% confidence in having all 16S variants from the same genome in the same group, a divergence of more than 0.03 must be allowed. This means clustering at no more than 97% identity.

**Table 2:** The table lists the 50%, 90%, 95% and 99% quantile of the predicted divergences for complete genomes across all species

Quantile (%)	Within genome	Within species	Within genus
50	0.0006	0.0008	0.0234
90	0.0041	0.0015	0.0349
95	0.0069	0.0022	0.0412
99	0.0306	0.0038	0.0581

The within genome values are divergences between variants of 16S within the same genome, within-species are between genomes within each species and within genus are between species within each genus

The fitted GLMM model revealed a small positive but significant effect of Chromosome level genomes on the within-genome divergence (around 3% increase to Complete Genome, *p*-value < 0.001). The estimated random-effect variances indicate the major variation in the data is at the lowest rank (species). It means within-genome divergence is not very tightly linked to the taxonomy but varies a lot even between genomes under the same species.

### Within taxon divergences

To establish how divergent the 16S are within the various ranks of the taxonomy, we computed divergences between pairs of taxa within their higher-ranking taxonomic category. From each genome (Complete Genome or Chromosome) only the most frequently occurring 16S variant was used. At the species rank, we computed divergences between genomes within each species. Then, between species within each genus, between genera within family, etc. up to the domain rank. In cases where we have many taxa, we down sampled to maximum 100 to avoid a too unbalanced data set. The down sampling was arranged by assembly level, i.e. we sampled randomly from the Complete Genomes first and then Chromosome until 100 sequences were selected.

Again, the pairwise divergences were used as response in the GLMM, as described for within-genome divergences above, the only difference being that the genome quality factor now has 3 levels. Since each divergence is between a pair of 16S sequences coming from different genomes, the sequences either come both from Complete genomes (Complete-Complete), both from Chromosome (Chromosome-Chromosome) or one from each (Complete-Chromosome). A GLMM was fitted to data for each rank separately, and predictions were made for the Complete-Complete factor level only. For the within-species data we employed the negative binomial model due to overdispersion, but for all other fitted models the Poisson was used. As seen from Table 3 the fitted GLMMs in most cases show the largest variance at the lowest rank. The estimated effects of Chromosome genomes on divergences were in all cases significant but small and in all cases positive, i.e. divergences tend to be slightly larger when comparing sequences from Chromosome quality genomes.

**Fig. 2:**
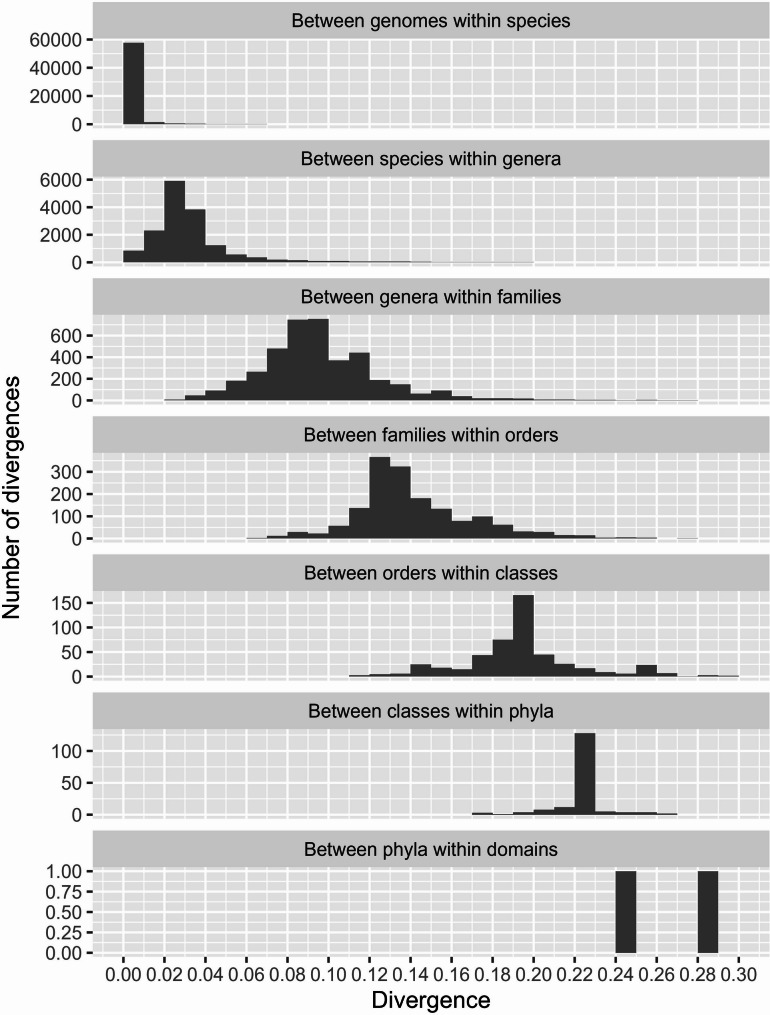
The histograms of predicted divergences between 16S from high-quality genomes based on the fitted GLMMs. All panels show predicted divergences (x-axes) nested within taxa at the various ranks. Note that the y-axes vary because the number taxa at the various ranks vary

In Fig. 2 we show how the predicted high-quality divergences from the fitted GLMMs distribute within each rank. The divergences within species are in general very small (< 0.01), and in fact not much larger than what we found between variants within a single genome. Between species, between genera, etc. the divergences grow, as expected. There are clearly large overlaps between the ranks, i.e. it is impossible to fix a single divergence threshold that will separate between all species and at the same time never split genera. The same applies at all higher ranks as well.

The second and third column in Table 2 list the predicted within- species and genus divergence for some quantiles for the high-high cases. As the within-genome predicted divergence these can serve as a guide to determining the level of stringency required when clustering 16S sequences at different levels of taxonomy to be confident that variants from the same species or genus fall into the same cluster. The difference between the three predicted levels increases as the quantile rises, and we can see that within-species always has the lowest divergence measure per quantile.

**Table 3:** Estimates for the taxonomy variances of the GLMM fitted to divergence within various taxonomic ranks

Parameter	Within species	Within genera	Within families	Within orders	Within classes	Within phyla
Species variance (*s*_*S*_^*2*^)	0.58	–	–	–	–	–
Genus variance (*s*_*G*_^*2*^)	0.14	0.68	–	–	–	–
Family variance (*s*_*F*_^*2*^)	0.09	0.14	0.46	–	–	–
Order variance (*s*_*O*_^*2*^)	0.06	0.08	0.24	0.64	–	–
Class variance (*s*_*C*_^*2*^)	0.07	0.03	0.09	0.09	0.30	–
Phylum variance (*s*_*P*_^*2*^)	0.04	0.06	0.15	0.27	0.70	> 0.99
Domain variance (*s*_*D*_^*2*^)	0.02	< 0.01	0.04	< 0.01	< 0.01	< 0.01

These are relative values, summing to 1.0 in each column. This makes it easier to see at which ranks we find the larger/smaller variances. The columns refer to different fitted GLMMs. In Within species the divergences were between genomes within species. In the remaining columns the divergences were between taxa within the rank named in the column. The results for Within domain are not listed, since all variation must then be at the domain rank (*σ*_*D*_^*2*^ *= 1.0*)

### Distinct sequence collection

The total set of 791 175 was reduced to a set of species-distinct sequences by finding, for each species, the subset of 16S sequences where all pairwise divergences are nonzero. This eliminates exact copies within the species, but also cases where a shorter sequence is a subsequence of a longer. In the latter case the longest sequence was retained. This resulted in a set of 201 279 sequences, and these are available as the supplementary file GTDB_16S_distinct_r226.tsv. Note that even if sequences are distinct within each species, they may be identical across species. We believe this collection is very useful for testing taxonomic classifications of 16S sequences using the GTDB taxonomy, and may also serve as a database for closed reference assignments of 16S reads.

### Clustering distinct sequences

**Fig. 3:**
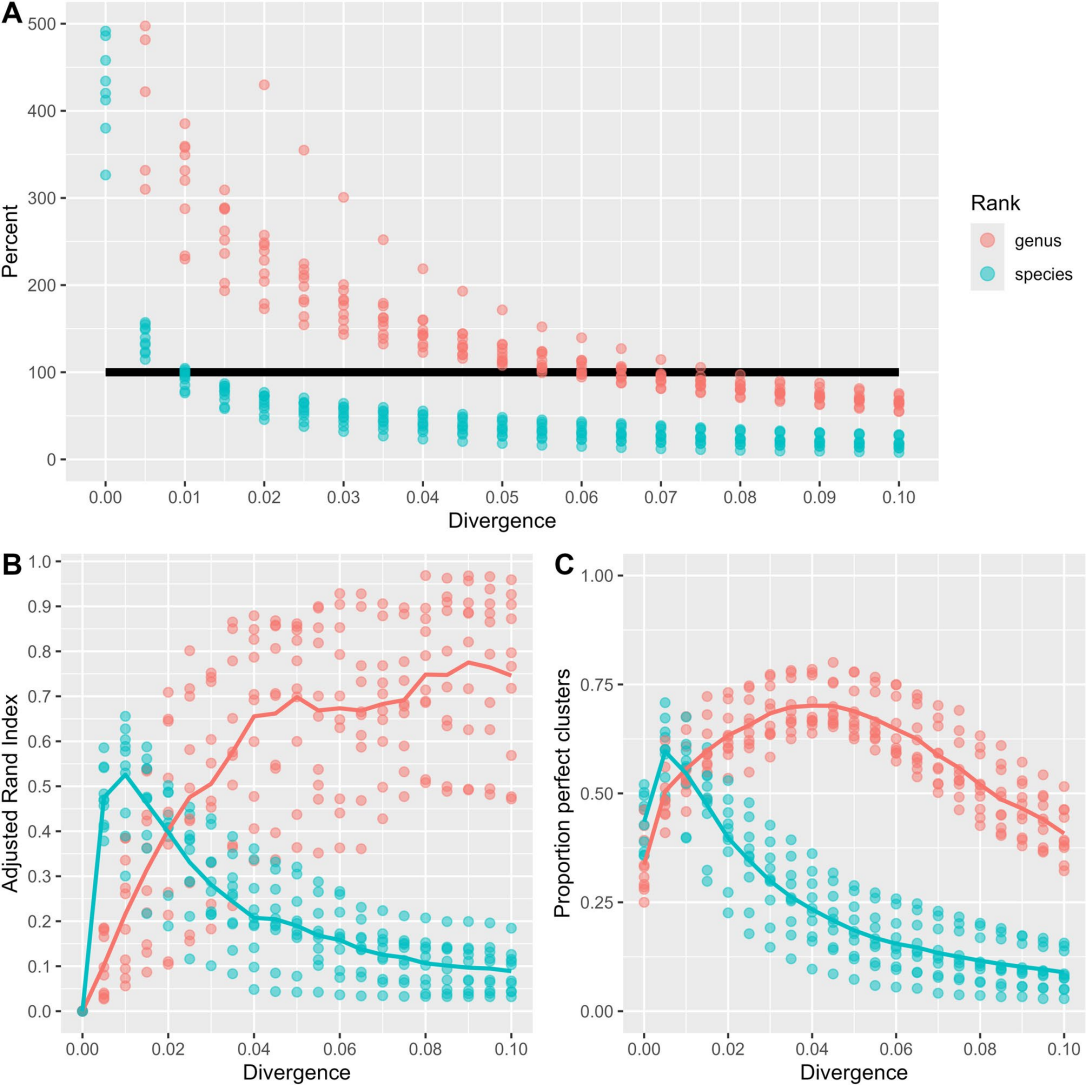
In the upper panel A we display the number of clusters (dots) as a percentage of the actual number of taxa for increasing divergence clustering thresholds (x-axis). The red dots are relative to the true number of genera for each subset and the blue dots relative to the true number of species. The 100% line is marked in black. The lower left panel B shows the Adjusted Rand Index (ARI) for each subset clustering at every divergence threshold (dots). Similarly, we see a corresponding plot in the lower right panel C, where the y-axis reflects the proportion of perfect clusters. The curves in panels B and C display the mean values of the ARI and the proportion of perfect clusters, respectively. Again, the red corresponds to comparing against true genus grouping and blue against the species grouping

Given the distinct sequences, we wanted to see how clustering them at various divergence/identity thresholds results in clusters corresponding to the grouping by actual species and genera for the sequences. From the distinct sequences we extracted only those from high quality genomes and no more than 100 distinct sequences from any species, resulting in 58 336 sequences. These were then split into subsets as described in Methods. Each subset was clustered at divergences from 0.0 to 0.1, i.e. from 100% to 90% identity. For each subset and divergence level we computed the number of clusters relative to the true number of species and genera for that subset. We also compared the actual grouping of the sequences to their true grouping by species or genus, using the Adjusted Rand Index (ARI) and the fraction of perfect clusters. A cluster is considered perfect at the species/genus level if it contains all sequences from some species/genus and nothing else. The results are displayed in Fig. 3. From panel A we see that the number of clusters is approximately the same as the number of genera when clustering at divergences around 0.06–0.07 (0.93–0.94 identity), i.e. this is where the red dots are closest to the 100% line. For species we get similar results at a divergence around 0.01. For these subsets the true number of genera varied around 300 and the true number of species around 1000.

From panel B we observe the best grouping (largest ARI) of the sequences with respect to genus is around 0.08–0.09, but there is a huge variation between the subsets. For species the clusters have a maximum ARI at 0.01 with a considerably lower variation. For panel C, we observe the same pattern as for the ARI, the proportion of perfect clusters for species peaks at 0.01, while the maximum at genus level is around 0.04.

**Fig. 4:**
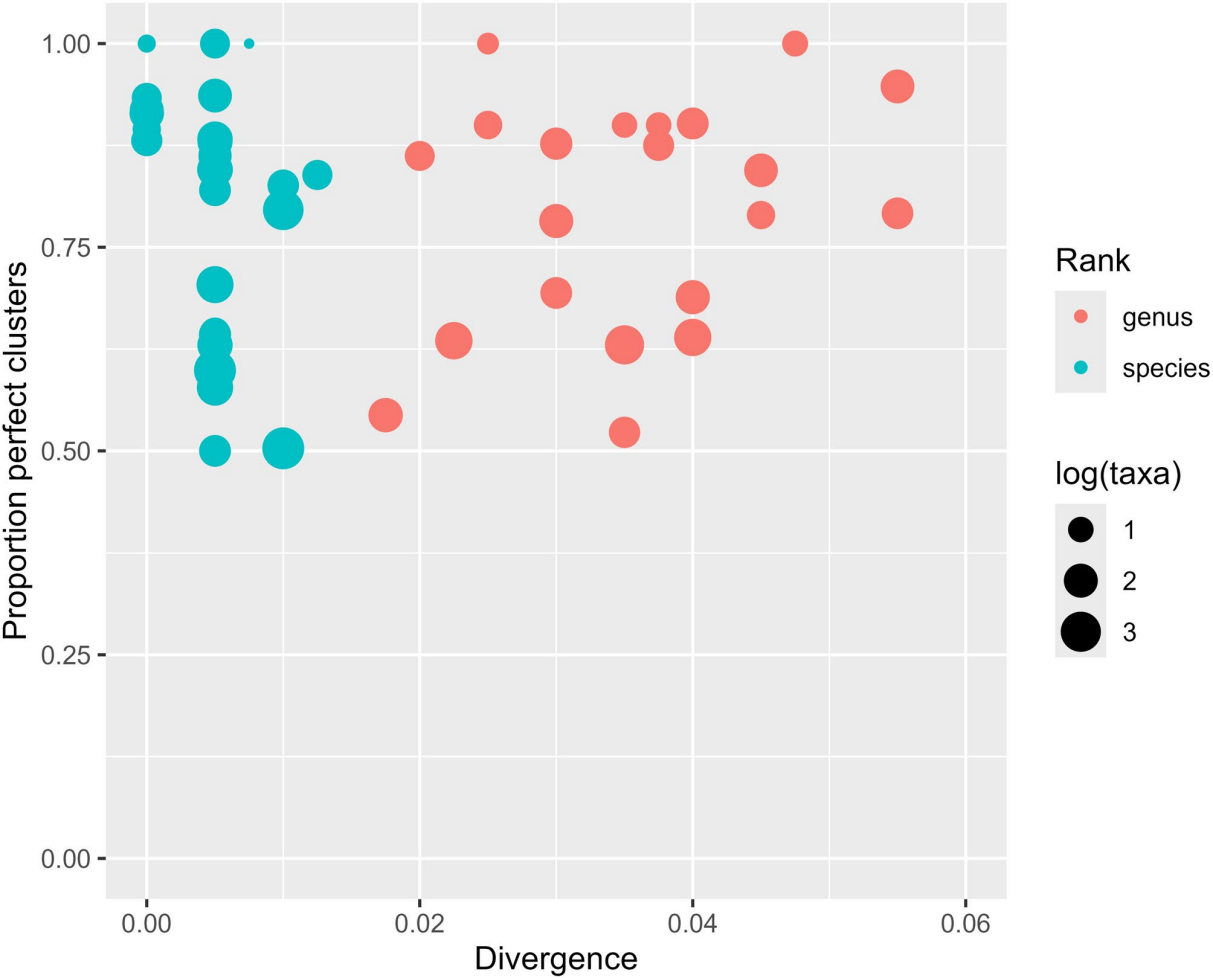
Each dot corresponds to a phylum and the position reflects the optimal clustering divergence (x-axis) and the proportion of perfect clusters (y-axis) when clustering the sequences within each phylum. The size of each dot reflects the log_10_ of the number of taxa (species/genera) in each phylum

From Figs. 1 and 2 we find there is quite some variation in how much 16S divergence we should expect to see within and between taxa. It is difficult to come up with one single divergence threshold that would work well all over. To illustrate this, we split all sequences by phylum and then did the clustering within each of these sets. For each phylum we then searched for the optimum divergence threshold by maximizing the proportion of perfect clusters relative to both species and genus grouping. Only larger phyla showing a distinct maximum are displayed, since phyla with very few taxa/sequences typically have ‘optimal’ clustering across a large divergence range. The results are shown in Fig. 4. By comparing this to panel C of Fig. 3, we can see that by choosing a phylum-specific divergence threshold, we increase the proportion of perfect clusters substantially. We also notice, especially for genus (red dots), how much the optimal divergence varies (x-axis) between phyla.

## Discussion

The GTDB initiative is a long-awaited effort to make the prokaryotic taxonomy more reproducible and consistent with the overwhelming amounts of genomic data we have today. We have in this study focused on the high-quality 16S marker gene variants extracted from the hundreds of thousand GTDB genomes and analyzed their divergence distribution in order to update our view on the 16S taxonomic resolution within this taxonomy. Our main findings are that in order to get a GTDB species resolution from 16S data we need to group such sequences by a divergence of around 0.01, i.e. a 99% sequence identity. For a genus resolution the corresponding values are around 0.05, i.e. 95% identity. These values can be directly compared to the widely used historical thresholds in prokaryotic taxonomy, where 97% identity has often been applied for species and 95% for genus [25, 26]. Our results thus suggest that the traditional 97% species threshold may underestimate diversity when evaluated against GTDB taxonomy, while the genus-level threshold of 95% is more consistent with our findings. Importantly, we also demonstrate that such thresholds vary considerably across different branches of the taxonomy, especially at the genus level, meaning that the use of a single universal cutoff is in general suboptimal.

Denoising of 16S reads has become a standard approach to processing such data (e.g [11, 12, 27]. The idea is to try to remove the ‘technical’ noise introduced by sequencing, resulting in the actual biological sequence variants. Our results (Table 2) show that genomes with multiple variants of the 16S have a within-genome divergence which is essentially at the same level as within-species. Thus, a denoising method that successfully eliminates all sequencing noise will result in distinct 16S variants that may very well be from the same strain. Thus, it makes little sense to only remove technical noise as long as we are faced with this ‘biological noise’ in 16S. A clustering at a divergence of at least 0.01 (99% identity) seems unavoidable to obtain biologically meaningful sequence variants. The ever-improving sequencing technologies means the technical noise will eventually be ignorable [14, 28]. But even with diminishing technical noise, biological noise will remain and will always require some degree of clustering.

Estimating 16S divergences from genomic data means we need to compensate for the quality of the genomes from which the 16S were extracted, as clearly seen in Fig. 1. The large divergences observed among 16S sequences from low-quality genomes highlight the challenges of reconstructing full-length 16S rRNA genes from short-read whole-genome assemblies [29]. Such discrepancies may arise from technical artifacts, including inclusion of sequences from closely related taxa, chimeric assemblies, misbinning during assembly, or poorly resolved scaffolding of fragmented contigs. These well-documented sources of error can substantially inflate the observed intra-genomic divergence values. For instance, if an assembly produces two or more contigs with 16S-like regions that differ by 15–30%, this should raise concern. Furthermore, it is striking that more than 44% of GTDB genomes lack any 16S gene. Labeling such genomes as ‘complete’ seems questionable, given the absence of the most widely used phylogenetic marker gene. Hopefully, genomes assembled from long-read technologies [30, 31] will improve on this, but still most genomes in our databases are from short-read data.

In this study, we used full-length 16S rRNA gene sequences as the basis for our analyses. Traditionally, many microbiome studies have focused on sequencing only selected regions of the 16S gene (most commonly the V3–V4 region). However, previous work has demonstrated that restricting analyses to variable regions can lead to a substantial loss of discriminatory power (e.g [14, 32, 33]). Recent advances in third-generation sequencing technologies such as PacBio and Oxford Nanopore have made full-length 16S rRNA gene sequencing increasingly accessible, providing higher taxonomic resolution compared to traditional region-specific amplicons (e.g., V3–V4) [34]. To examine how genome-based taxonomy provides insights into 16S divergence within and between taxa at all ranks, we consider it appropriate to analyze the entire gene, as this approach provides the most comprehensive resolution.

The expected within taxon divergence is the information we need in order to cluster 16S sequence variants into biologically meaningful groups. We fitted the GLMM to data at all ranks from species to phylum. In all cases the divergences are only between taxa, e.g. between species within the genus they belong to. The predicted divergences we get from this model is the expected divergence we would see for high-quality genomes only.

From Table 2 and the upper two panels of Fig. 2 we can see it is in general challenging to separate between GTDB-defined species based only on 16S data even if these are near full-length and essentially error free sequences obtained from high-quality genomes. A divergence threshold of at least 0.01 is required to ensure the majority of sequences from the same species are grouped together (upper panel), while the second panel shows a substantial fraction of divergences between species is below this value. A species, and even strain-level, resolution may be possible [14] if the samples are from microbiomes where we already have databases of high quality 16S variants (e.g. the human gut) and could use a closed-reference assignment, but for environmental studies this is rarely the case. For such studies a species resolution requires long reads with precision well over 99%.

The same overlap applies to all ranks in Fig. 2, i.e. it is impossible to use a single fixed threshold that will separate well between all taxa at one rank but not split taxa at the rank above. It should also be noted that for larger divergences (lower panels of Fig. 2) the Hamming distance we have used here (percent identity) is under-estimating the actual evolutionary distances [35]. Correcting with some evolutionary model would increase all values, and lead to even wider histograms.

The clustering of the sequences we obtain from the high-quality genomes only confirm the results we got from the predicted divergences, see Fig. 3. In order to get clusters who are closest to a species grouping a divergence of around 0.01 is optimal, while in order to obtain a genus-like clustering divergence thresholds of 0.04–0.08 are needed.

Perhaps the most striking result is the substantial variation in optimal divergences between phyla, as seen in Fig. 4. When comparing the clustering to the actual grouping by genus (red dots) we find the optimal divergence thresholds vary from 0.02 to more than 0.06. Also, it is easier to classify Phylum with few taxonomic groups than with more diverse taxa. This is not very surprising, but worth noting in terms of classifying real, complex data. If we can classify reliably at the phylum level, knowledge of complexity within the phylum can give us an indication of how confident we can be in classification further down the hierarchy. We believe these results, along with our predicted divergences for all taxa at all ranks, paves the way for a new approach to analyzing environmental 16S data. Even for environments poorly represented in the databases, we can still make fairly reliable taxonomic assignments to phylum or some other higher rank, and then adjust clustering thresholds accordingly. This is something that should be pursued in order to make more reliable estimates of taxonomic diversity from environmental data.

## Supplementary Information

Below is the link to the electronic supplementary material.

Supplementary Material 1

## Data Availability

No datasets were generated or analysed during the current study.
